# Contribution of 100% Fruit Juice to Micronutrient Intakes in the United States, United Kingdom and Brazil

**DOI:** 10.3390/nu12051258

**Published:** 2020-04-28

**Authors:** Ellen S. Mitchell, Kathy Musa-Veloso, Shafagh Fallah, Han Youl Lee, Peter J. De Chavez, Sigrid Gibson

**Affiliations:** 1 Nutrition Sciences, PepsiCo R&D, Purchase, New York, NY 10577, USA; 2 Intertek Health Sciences Inc., Mississauga, ON L5N 2X7, Canada; 3 Data Science and Analytics, PepsiCo R&D, Barrington, IL 60010, USA; 4 Sig-Nurture Ltd., Guildford, Surrey GU1 2TF, UK

**Keywords:** juice, dietary modeling, nutrients, dietary survey, Pesquisa de Orçamentos Familiares, National Diet and Nutrition Survey, National Health and Nutrition Examination Survey

## Abstract

The contribution of 100% fruit juice (FJ) to the total daily intakes of energy, sugars, and select vitamins and minerals and to the recommended dietary allowances (RDAs) or adequate intake (AI) of these micronutrients was assessed in individuals reporting the consumption of 100% FJ in the national dietary intake surveys of the United States (U.S.; *n* = 8661), the United Kingdom (UK; *n* = 2546) and Brazil (*n* = 34,003). Associations of 100% FJ intake with the odds of being overweight or obese also were assessed. Data from the U.S. National Health and Nutrition Examination Survey (2013–2014), the UK National Diet and Nutrition Survey (2012–2014), and Brazil’s Pesquisa de Orçamentos Familiares (2008–2009) were used, and all analyses were limited to individuals reporting consumption of 100% FJ on at least one day of the dietary intake survey. Approximately 34%, 37%, and 42% of individuals surveyed reported the consumption of 100% FJ on at least one day of the dietary intake survey in the U.S., UK, and Brazil, respectively, and the average daily intakes of 100% FJ were 184 g, 130 g, and 249 g, respectively. Across the 3 countries, 100% FJ contributed to 3–6% of total energy intakes, 12–31% of total sugar intakes, 21–54% of total vitamin C intakes, 1–12% of total vitamin A intakes, 4–15% of total folate intakes, 7–17% of total potassium intakes, 2–7% of total calcium intakes, and 4–12% of total magnesium intakes. In a multivariate logistic regression model, juice intake was associated with a significant reduction in the odds of being overweight or obese in UK adults (OR = 0.79; 0.63, 0.99), and significant increases in the odds of being overweight or obese in UK children (OR = 1.16; 1.01, 1.33) and Brazilian adults (OR = 1.04; 1.00, 1.09). Nutrient contributions of 100% FJ vary according to regional intake levels. In all three countries studied, 100% FJ contributed to more than 5% of the RDAs for vitamin C and folate. In the U.S. and Brazil, 100% FJ contributed to more than 5% of the RDA for magnesium and more than 5% of the AI for potassium.

## 1. Introduction

One of the most important goals in public health nutrition is ensuring adequate nutrient intakes and diet quality across all age groups within a population. In developed countries, one of the distinct challenges in adhering to a nutritionally adequate diet is achieving dietary recommended intakes without exceeding energy requirements, via the selection of foods that contain an appropriate balance of nutrients versus calories. One food group that has increasingly come under scrutiny is 100% fruit juice (FJ). Historically, 100% FJ has been perceived as a convenient source of nutrients, such as vitamin C and potassium; yet, more recently, 100% FJ has been criticized as a vehicle that delivers free sugars and excess energy, especially for children. The question of whether the consumption of 100% FJ leads to weight gain has been the subject of several research papers.

In a meta-analysis of longitudinal studies on the association between FJ consumption and BMI in children aged 1 to 18 years, Auerbach et al. reported a statistically significant but clinically irrelevant increase in BMI z-score in children aged 1 to 6 years, and no association in children aged 7 to 18 years [1]. Using data from the 2001 United States (U.S.) nationally representative Early Childhood Longitudinal Study—Birth Cohort, Shefferly et al. reported that the regular consumption of 100% FJ at 2 years of age was associated with a significant increase in the odds of being overweight between 2 and 4 years of age [2]. No association between FJ consumption and overweight or obesity was reported in several other observational studies, most of which were cross-sectional in design [3,4,5,6]. For example, using data for children 2 to 18 years of age from the 2003 to 2006 U.S. National Health and Nutrition Examination Survey (NHANES), O’Neil et al. reported no significant difference in the odds of being overweight or obese among consumers and non-consumers of 100% orange juice; though, consumers of 100% orange juice had a significantly greater Healthy Eating Index than non-consumers, with a greater percentage of orange juice consumers meeting the estimated average requirement (EAR) for vitamin A, vitamin C, folate, and magnesium [3]. In adults, based on nationally representative dietary intake data collected in France as part of the “Comportements et Consommations Alimentaires en France” (CCAF) study, it was reported that FJ consumption was not associated with BMI; though, 100% FJ contributed to the intakes of several vitamins (B1: 7%, B2: 3%, B5: 5%, B6: 6%, B9: 10%, C: 32%, E: 9%, beta-carotene: 5%) and minerals (magnesium: 4%, potassium: 7%), as well as free sugars (19%) [4].

Overall, in studies conducted in the U.S., UK, and France, it was reported that 100% FJ intake is not associated with increased BMI, neither in adults nor children, but is associated with healthier diets and lifestyles, higher education and economic status, and improved metabolic risk factors, such as antioxidant status and insulin sensitivity [3,4,5,6]. The relationship between 100% FJ intake and BMI in Brazilians has not yet been published, although it was reported by Xavier et al. that the prevalence of occasional 100% FJ consumption among Brazilian adolescents was 37.6% and 32.1% in rural and urban areas, respectively [7].

The U.S., the UK, and Brazil are geographically distinct regions with different agricultural emphases and cultural practices, which, in turn, influence overall dietary habits and quality, i.e., nutrient gaps as well as daily energy intake. In both the U.S. and UK, lower than recommended dietary intakes have been reported for several nutrients that are typically found in 100% FJ. Specifically, in the U.S., there is evidence of a prevalence of inadequate intakes for vitamin C (25% below EAR), vitamin A (34% below EAR), potassium (97% below AI), magnesium (45% below EAR), and calcium (38% below EAR) [8]; while, for the UK, the Summary of Key Findings from the NDNS Report of Years 7 and 8 highlighted that folate, potassium, and calcium intakes were below the lower reference nutrient intakes (LNRI) for particular age groups, such as teenage girls (below LNRI: 15%, 22%, 38% respectively) [9] In the two-day national dietary intake survey for Brazil, the Pesquisa de Orçamentos Familiares (POF 2008–2009), the prevalence of inadequate intakes for calcium and vitamins A, C, D, and E exceeded 15% in males and females aged eleven years and older, though intakes of potassium were sufficient [10]. In a recent study, it was reported that the main dietary pattern of Brazil, which includes traditional beans and rice, is being supplanted by a “Western” pattern of fried foods and sweet snacks, and this will likely alter nutrient intakes [11].

The objective of the study reported herein was to assess the overall consumption patterns and nutrient contributions of 100% FJ in three countries of varying geographies, cultures, socioeconomic levels, and dietary habits; namely, the U.S., the UK, and Brazil. Intakes of 100% FJ also were assessed with regard to age, gender, and BMI. While many groups have previously reported 100% FJ consumption in one region or country, few have examined 100% FJ consumption and its nutritional contributions in the context of the overall diet in multiple countries. The differential role 100% FJ plays in delivering nutrients to specific populations, with regard to regional juice preferences and overall nutrient intake, also was examined.

## 2. Materials and Methods

### 2.1. Nutrition Surveys

Three nationally representative dietary intake surveys were used in the analyses presented herein: the U.S. 2013–2014 NHANES, the UK 2012–2014 National Diet and Nutrition Survey (NDNS), and Brazil’s 2008–2009 POF.

The NHANES is a nationally representative, cross-sectional survey that samples the U.S. civilian population ages birth to 90 years old, using a complex, stratified, multistage probability cluster sampling design. The NHANES data are collected by the National Center for Health Statistics of the U.S. Centers for Disease Control. Written informed consent was obtained from all participants or proxies and the survey protocol was approved by the Research Ethics Review Board at the National Center for Health Statistics. In the 2013–2014 NHANES, 8661 individuals were surveyed for sociodemographic, dietary, and medical history information. NHANES participants were asked to complete a health examination and 24-hour dietary recall via in-person interview, followed by a second 24-hour dietary recall collected via telephone, ∼3–10 d later. For both 24-hour recalls, proxy respondents reported for children who were 5 years and younger and proxy-assisted interviews were conducted with children 6–11 years of age. 

For the UK NDNS, a four-day food diary was completed by the survey participants. Measurements of height and weight were collected during the survey period. For the 2012–2014 UK NDNS, complete food diaries (i.e., for at least 3 of the 4 days) were available for 2546 individuals aged 1.5 years and older. THE NDNS is nationally representative and dietary intake data are collected on a rolling basis.

The Brazilian POF has a similar design to NHANES, with two non-consecutive 24-hour dietary recalls conducted via interview, and the additional collection of medical, social and economic information. For the 2008–2009 Brazil POF, intake data were available for 34,003 individuals aged 10 years or older.

### 2.2. Analyses

Included in the analyses were those individuals who reported the consumption of any amount of 100% FJ on at least one day of the dietary survey (i.e., on either Day 1 or Day 2 of NHANES or POF, and either Day 1, 2, 3, or 4 of NDNS). Excluded from the analyses were breastfeed babies, pregnant and lactating women, individuals with incomplete dietary records, and, for the U.S., individuals with only 1 day (as opposed to 2 days) of dietary intake data. For the U.S. NHANES, it was possible to remove individuals with only 1 day of dietary intake data, given that 1-day and 2-day sample weights are provided; however, this was not the case for the Brazil and UK surveys, and so individuals who did not have dietary intake data for the entire length of the survey were maintained in the analyses for Brazil and the UK. For the UK survey, all individuals had at least 3 days of complete diet records. For the Brazil POF, 1103 out of 34,003 individuals (3.2%) had only 1 day of dietary intake data, as opposed to 2 days.

Nectars and sugar-sweetened fruit drinks were not defined as 100% FJ in the present analysis. We defined 100% FJ as drinks categorized as having a 100% FJ content, including mixtures of juices (See Supplementary Table S1, Table S2 and Table S3 for full listings of food codes used for the U.S., UK, and Brazil analyses, respectively). The numbers of relevant food codes for 100% FJ in the 2013–2014 NHANES, the 2012–2014 NDNS, and the 2008–2009 POF were 53, 31, and 15, respectively.

Using data from each survey, we calculated the intakes of 100% FJ, as well as the contribution of 100% FJ to the intakes of total energy, total sugars, and select vitamins and minerals (i.e., vitamin C, vitamin A, folate, potassium, calcium, and magnesium). The intakes of total energy, total sugars, and total vitamin C, vitamin A, folate, potassium, calcium, and magnesium were based on the consumption of foods and beverages (including water) but do not include intakes from medications or dietary supplements. For the vitamins and minerals, we also calculated the contribution of 100% FJ to the National Academy of Medicine (NAM) age- and gender-specific recommended dietary allowances (RDAs) or adequate intakes (AIs; in the case of potassium). The intakes of 100% FJ, as well as the contribution of 100% FJ to the intakes of total energy, total sugars, and total vitamin C, vitamin A, folate, potassium, calcium, and magnesium as well as to the NAM RDAs (or AI, in the case of potassium) for these nutrients were calculated by averaging intakes over the total number of survey days. For each intake assessment, results were tabulated according to gender and for different age groups, data permitting [infants (<1.5 years, U.S. only); toddlers (1.5 to 2 years, U.S. and UK); children (3 to 9 years, U.S. and UK); children (10 to 12 years, all surveys) adolescents (13 to 19 years, all surveys); young adults (20 to 59 years, all surveys); older adults (60 years+, all surveys). In addition, using the number of consumers of each 100% FJ, we identified, from each survey, the most popular of each 100% FJ category that was consumed by children and adults.

Using a multivariate logistic regression model, the associations between consumption of 100% FJ (treated as a continuous variable) and the odds of being overweight/obese were assessed separately amongst adults and children identified as consumers of 100% FJ. For the analyses for each country, BMI status was grouped into two categories: (i) underweight or normal weight; and (ii) overweight or obese, as has been done by others in similar assessments [12]. In the UK NDNS, the BMI status of adults 20 years of age and older is provided as “1”, “2”, “3”, “4”, or “5”, corresponding to BMI classifications of underweight (<18.5 kg/m^2^), normal weight (18.5–24.9 kg/m^2^), overweight (25–29.9 kg/m^2^), obese (30–39.9 kg/m^2^), and morbidly obese (≥40 kg/m^2^), respectively. Amongst adults identified as 100% FJ consumers, we combined into one category all underweight and normal weight adults (i.e., normal weight or underweight), and combined, into another category, all overweight, obese, and morbidly obese adults (i.e., overweight or obese). In the UK NDNS, body weight status for children 2 to 19 years of age is provided as “1”, “2”, or “3”, corresponding to normal weight or underweight (BMI < 85th percentile), overweight (BMI in the 85th to < 95th percentiles), or obese (BMI ≥ 95th percentile), respectively (based on the UK’s and the World Health Organization’s (WHO’s) growth chart data). We combined overweight and obese children into a single category (i.e., overweight or obese).

In the U.S. NHANES, the BMI status of children aged 2 to 19 years is classified as underweight (BMI < 5th percentile); normal weight (BMI 5th to < 85th percentiles); overweight (BMI 85th to < 95th percentiles); or obese (BMI ≥ 95th percentile). Cutoff criteria were based on the Centers for Disease Control and Prevention’s sex-specific 2000 BMI-for-age growth charts for the U.S. Age in months at examination was used to match age in months from BMI growth chart data, separately for males and females. Adult BMIs were classified according to the WHO criteria (i.e., underweight (<18.5 kg/m^2^), normal weight (18.5–24.9 kg/m^2^), overweight (25–29.9 kg/m^2^), or obese (30–39.9 kg/m^2^)).

For the Brazil survey, we computed BMI from height and weight (weight (kg)/height (m)^2^), which was measured during survey interviews. For adults (defined as aged 20 years or older), values between 18.5 and 25 kg/m^2^ were categorized as normal weight, while overweight and obese were defined as BMI values > 25 and <30 kg/m^2^ and ≥30 kg/m^2^, respectively. For Brazilian children, BMI categorizations were made using WHO standard reference data, where overweight was classified as >+1SD (equivalent to BMI 25 kg/m^2^ at 19 years), obesity was classified as: >+2SD (equivalent to BMI 30 kg/m^2^ at 19 years), and normal weight was classified as <+1SD. For each country, participants with BMI values that were classified as underweight or normal weight were combined into a single reference category (i.e., underweight or normal weight). Overweight and obese individuals also were combined into a single category (i.e., overweight or obese).

Covariates included in the multivariate logistic regression model were the amount of 100% FJ consumed, age, gender (males versus females), race (white versus others), and income, which was defined as the ratio of family income to poverty for the U.S. NHANES, the equivalized household income for the UK NDNS, and total family income for the Brazilian POF. The multivariate logistic regression model was run with and without total daily energy intake. The covariates were chosen based on what has been included in other studies [2,3,4]. For all surveys, individuals identified as consumers of 100% FJ, but for whom BMI or covariate data were missing, were excluded from the assessment of the association of 100% FJ consumption and odds of being overweight or obese. Of note, BMI classifications in the U.S. and UK datasets are provided only for children 2 years of age and older; hence, infants and toddlers were not included in the multivariate logistic regression models. Statistical significance was defined as a P-value < 0.05, and all P-values were two-tailed. Sampling weights, primary sampling units, and strata were used for weighting in all statistical analyses. SAS (Version 9.4, SAS Institute Inc., Cary, NC, USA) was used to carry out all statistical analyses.

## 3. Results

**Figure 1:**
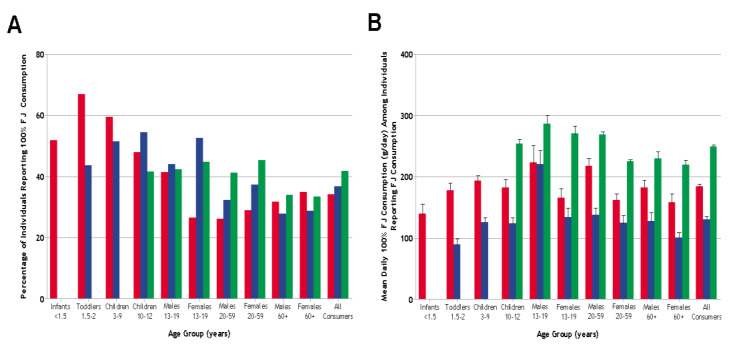
(**A**) Percentage of individuals reporting consumption of 100% fruit juice (FJ); (**B**) Average daily intakes (mean ± SEM) of 100% FJ (g/day) in individuals reporting consumption of 100% FJ. The “all consumers” category does not include infants <1.5 y for the UK and children <10 y for Brazil, as these individuals were not included in the respective surveys. Red bars = U.S.; blue bars = UK; green bars = Brazil.

The percentage of individuals reporting the consumption of 100% FJ was 34% in the U.S., 37% in the UK, and 42% in Brazil (see Figure 1). Among individuals reporting the consumption of 100% FJ, the daily intakes of 100% FJ (averaged over the lengths of the dietary surveys) were 184, 130, and 249 g in the U.S., the UK, and Brazil, respectively. One hundred percent FJ provided an average of 88 kcal/day (4.7% of total energy), 50 kcal/day (3.0% of total energy), and 109 kcal/day (6.2% of total energy) in the U.S., UK, and Brazil, respectively. The average intakes of sugars from 100% FJ in the U.S., the UK, and Brazil were 17, 12, and 21 g/day, respectively, accounting for 15%, 12%, and 31% of total sugar intakes, respectively (see Table 1).

U.S. individuals reporting the consumption of 100% FJ consumed an average of 51.4 mg/day of vitamin C, 2.7 mcg/day of vitamin A, 20.0 mcg/day of folate, 267.9 mg/day of potassium, 77.8 mg/day of calcium, and 16.3 mg/day of magnesium from 100% FJ. Individuals reporting the consumption of 100% FJ in the UK had average intakes of 33.0 mg/day vitamin C, 5.5 mcg/day vitamin A, 22.3 mcg/day of folate, 180.4 mg/day of potassium, 12.8 mg/day of calcium, and 10.1 mg/day of magnesium from 100% FJ. In Brazil, in individuals reporting the consumption of 100% FJ, 100% FJ accounted for the intakes of 267.8 mg/day of vitamin C, 31.7 mcg/day vitamin A, 41.1 mcg/day folate, 440.3 mg/day potassium, 20.0 mg/day calcium, and 29.0 mg/day of magnesium (see Table 1).

**Figure 2:**
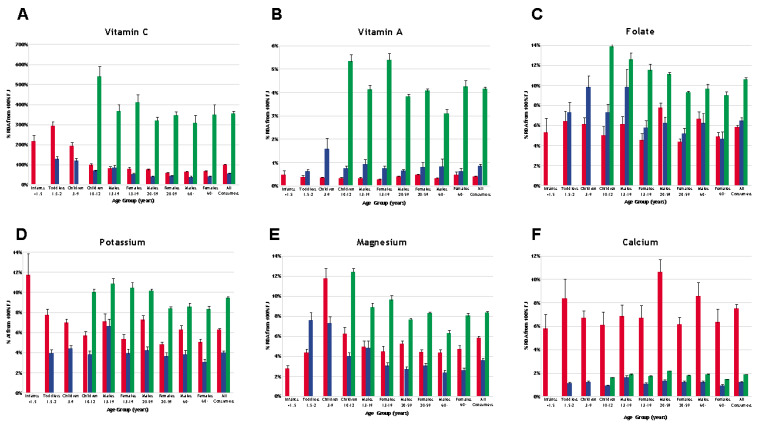
Percentage recommended dietary allowance (RDA) of vitamin C (**A**), vitamin A (**B**), folate (**C**), potassium (**D**), magnesium (**E**), and calcium (**F**) contributed by 100% FJ in individuals reporting the consumption of 100% FJ in the U.S. (red bars), UK (blue bars), and Brazil (green bars) (mean + SEM). Note, the “all consumers” category does not include infants <1.5 years for the UK and children <10 years for Brazil, as these individuals were not included in the respective surveys.

In U.S. individuals reporting the consumption of 100% FJ, 100% FJ provided more than 5% of the RDA for vitamin C, folate, magnesium, and calcium, and more than 5% of the AI for potassium. In UK individuals reporting the consumption of 100% FJ, 100% FJ provided more than 5% of the RDA for vitamin C and folate. In Brazilian individuals reporting the consumption of 100% FJ, 100% FJ provided more than 5% of the RDA for vitamin C, magnesium, and folate, and more than 5% of the AI for potassium (see Figure 2A–F). Apple juice was the most popular 100% FJ among U.S. children, while orange juice was the most popular 100% FJ among U.S. adults. In the UK and Brazil, orange juice was the most popular 100% FJ, both for adults and children.

The numbers (and percentages) of consumers of 100% FJ identified as underweight, normal weight, overweight, or obese in each of the three surveys are summarized in Table 2. As can be seen in Table 2, the percentage of individuals classified as underweight was slightly larger among children than adults, but was less than 4% for all survey populations. Among individuals reporting the intake of 100% FJ, the proportion falling in the overweight or obese category was greatest in the U.S., followed by the UK, and then Brazil, for both children and adults.

Results of the multiple logistic regression models are summarized in Table 3, Table 4, and Table 5 for the U.S., UK, and Brazil, respectively. For each survey, results from the model that included energy intake were generally similar to those from the model that did not include energy intake. Older age among adults was associated with a significant increase in the odds of being overweight or obese for all three surveys. In children aged 10 to 19 years in Brazil, older age was associated with a significant decrease in the odds of being overweight or obese. Gender (i.e., being a male) was associated with a significant increase in the odds of being overweight or obese among adults in the U.S. and Brazil surveys and among children in the Brazil survey. Being white was associated with a significant decrease in the odds of being overweight or obese among children in the U.S. survey and among adults in the UK survey. Greater income was associated with a significant decrease in the odds of being overweight or obese in children in the UK survey, but a significant increase in children in the Brazil survey. Energy intake was not associated with the odds of being overweight or obese in any of the surveys, neither for children nor adults. Amount of 100% FJ consumed was not associated with the odds of being overweight or obese in the U.S. survey, neither for children nor adults. Using data from the UK survey, a greater intake of 100% FJ was associated with a significant decrease in the odds of being overweight or obese in adults, and a small but significant increase in the odds of being overweight or obese in children. Using data from the Brazil POF, a greater intake of 100% FJ was associated with a small but significant increase in the odds of being overweight or obese in adults; however, in the model which did not include energy, the association of amount of 100% juice consumed and odds of being overweight or obese trended toward significance (*P* = 0.07).

## 4. Discussion

Using nationally representative dietary intake surveys, the daily intake of 100% FJ and the contribution of 100% FJ to the intakes of several nutrients and also the percent RDAs for these nutrients in the U.S., the UK, and Brazil were assessed. Across the different age groups, the prevalence of 100% FJ consumption ranged from 26–67% in the U.S., 27–54% in the UK, and 33–45% in Brazil. Compared to individuals reporting consumption of 100% FJ in the U.S. and UK, those in Brazil reported greater intakes of 100% FJ, and thus higher intakes of sugars and vitamins and minerals from 100% FJ (except for calcium, which is not naturally present at high levels in 100% FJ, but is used as a fortificant in 100% FJs in the U.S., hence the higher intakes of calcium from 100% FJ in the U.S. relative to the UK and Brazil).

Observations of differences between countries in the intakes of 100% FJ and the contribution of 100% FJ to the intakes of different nutrients must take into account the methodologies used in each national dietary intake survey. The UK NDNS has food records for four days, while the U.S. NHANES and Brazil POF are based on two, 24-hour diet recalls. Included in the analyses presented herein were individuals reporting any amount of 100% FJ on at least one day of the dietary survey. It is expected that there are individuals who consume 100% FJ infrequently, in which case the participants may not have been identified as 100% FJ consumers in our analyses, due to the limited number of intake days captured for each participant in each of the surveys. Likewise, if 100% FJ was consumed episodically only, as opposed to everyday, then the estimate of the daily intake of 100% FJ in a dietary intake survey spanning several days would be lower than that estimated in a survey spanning only 1 or 2 days, given that the daily intake of 100% FJ for each consumer was estimated by summing the intakes of 100% FJ across all survey days and dividing by the total number of survey days. Indeed, the mean intake of 100% FJ was lower in the UK (which had the highest number of survey days) than in the U.S. and Brazil.

To understand the impact of dietary survey length on intakes of 100% FJ, we assessed intakes of 100% FJ in individuals reporting consumption on only Day 1 of the 2-day NHANES, as well as those who consumed 100% FJ on both days of the 2-day NHANES, and compared these results to values reported herein (i.e., averages over the 2 days of the survey). These analyses could not be undertaken for the UK and Brazil, given that separate sample weights for each day of the surveys are not provided, as they are for NHANES. As can be seen in Supplementary Table S4, approximately 35% of individuals identified as consumers of 100% FJ consumed 100% FJ on both days of the dietary survey, with a mean intake of 280.7 g/day. Approximately 32% of individuals identified as consumers of 100% FJ consumed 100% FJ only on Day 1 of the 2-day survey, with a mean intake of 244 g/day. The remainder (33%) consumed 100% FJ only on Day 2 of the 2-day survey (the mean intake for these individuals could not be calculated, given that sample weights for NHANES are provided only for Day 1 or both Days 1 and 2, but not Day 2). Reported herein are the intakes of 100% FJ averaged over the entire length of the survey (for NHANES, 184 g/day). Thus, clearly, the length of the dietary survey will affect the estimated intake of 100% FJ, particularly if 100% FJ is not consumed daily. 

Another important difference between the dietary intake surveys in the U.S., the UK, and Brazil is the tool used to collect dietary intake data. In the U.S. and Brazil dietary intake surveys, subjects were asked to recall, on two different days, what they consumed in the previous 24 h; in contrast, in the UK dietary intake survey, subjects were asked to record foods and beverages consumed based on portion sizes estimated from photos and household measures. All dietary intake surveys are associated with limitations. For instance, in providing 24-hour diet recalls, participants may not remember what they consumed or may inaccurately report (either accidentally or intentionally) the types and amounts of foods consumed [13]. While food records are intended to provide more accurate information, they are more burdensome than 24-hour diet recalls, and the accuracy of the information collected is completely dependent on the participants’ dedication and motivation to collect and record the weighted information in real-time. Given the need to weigh food items consumed, participants may alter their dietary intakes during the survey days (e.g., the subjects cannot easily weigh foods consumed while eating out, and so may eat out less than if they were not taking part in the survey). Irrespective of the method used to collect dietary intake data, the under-reporting of energy and several micro- and macronutrients is a recognized problem, particularly in females, and particularly in participants who are either overweight or obese, as reviewed by Ahluwalia et al. [14]. Thus, rather than comparing differences between the countries in the intakes of 100% FJ, it is more appropriate to assess and compare the nutritional impact of juice consumption among those identified as consumers in each of the three countries.

Using data from adults enrolled in the 2003–2006 U.S. NHANES cycles, it was reported that a higher percentage of consumers of orange juice met the EARs for vitamin A, vitamin C, folate, and magnesium, as well as the AI for potassium, compared with non-consumers of 100% orange juice [15]. In the current study, we observed that 100% FJ provided U.S. adults with 37%, 7%, and 10% of the total daily intakes of vitamin C, calcium, and potassium, respectively. Calcium intakes from 100% FJ are likely due to the fortification of many juices with calcium (often in combination with vitamin D). Drinking fortified juice has become an increasingly popular method to obtain these nutrients, since Americans are moving toward plant-based diets and are consequently drinking less dairy milk. One hundred percent orange juice enriched with calcium and vitamin D is the most commonly consumed fortified 100% FJ; per serving, it contains 375 mg calcium, negligible fiber, 500 mg of potassium and 124 mg vitamin C, as reported in the USDA database of food products [16]. A study which modeled substitution of whole fruits in place of FJ concluded that average daily 100% FJ intake provided higher daily amounts of vitamin C, calcium and potassium than daily intake of whole fruits, and also noted FJ was more cost-effective and convenient; however, 100% FJ also was associated with increased intakes of energy and decreased intakes of fiber relative to whole fruit [17]. In the 2015–2020 Dietary Guidelines for Americans (DGA), the recommended intake of fruits is 2-cup equivalents per day, and 1 cup of 100% FJ is counted as 1 fruit-cup equivalent [18]. In the DGA, it is explicitly stated that “Although fruit juice can be part of healthy eating patterns, it is lower than whole fruit in dietary fiber and when consumed in excess can contribute extra calories. Therefore, at least half of the recommended amount of fruits should come from whole fruits” [18].

In the most recent NDNS survey (2015–2016) vitamin A, folate, potassium, calcium, and magnesium were reported as nutrients of concern for children and women [9]. One hundred percent FJ contributed to more than 5% of the RDA only for folate and vitamin C. UK consumers appear to have lower intakes of 100% FJ, though this could be an artifact of a longer dietary intake survey (and therefore a larger denominator used in calculating average intakes). Of note, there are fewer fortified juices available in the UK.

Published data on the intakes of 100% FJ in Brazil are scarce. A recent study of Brazilian beverage consumption in the 2008–2009 POF reported that, across ages, caloric coffee contributed the most calories, followed by sugar sweetened beverages, followed by juice [19]. The overall rate of juice consumption appears to be similar in rural versus urban areas and across age groups, but correlated to higher income [19]. Also, it was reported that in children who consume 100% FJ, on average, they consume 100–140 mL/day, out of the 2 L of liquids consumed daily [20]. Brazilian diets tend to be less diverse than diets in the U.S. or UK, and also exhibit more shortfalls in micronutrients, particularly vitamins A, C, D, and E, and calcium, potassium, iron and folate [10]. While our analysis showed a very high intake of vitamin C from 100% FJ, previous studies have reported that over 50% of Brazilian adults have insufficient intakes of vitamin C [10]. It is presently unclear what proportion of non-consumers of juice are among those 50% who have insufficient vitamin C intakes (<90 mg/d, men; 75 mg/d, women). It also must be noted that specific types of juice popular in Brazil, such as acerola (ranked as the third most popular juice in our analyses), are quite high in vitamin C, up to 700 mg/serving, and thus acerola juice drinkers are likely skewing mean intakes. On the whole, 100% FJ in Brazil tends not to be fortified, although, recently a trial using iron-fortified orange juice decreased the prevalence of iron deficiency anemia in preschool children [21]. Of course, orange juice is high in vitamin C, and it is well-known that vitamin C facilitates the absorption of iron, and so it is an ideal matrix for iron fortification.

Sugar contributions from 100% FJ averaged 15%, 12%, and 31% of total sugar intakes in individuals reporting consumption in the U.S., UK, and Brazil surveys, respectively. The contribution of 100% FJ to total sugar intakes in Brazil is higher than in the U.S. or UK, and this is a function of a greater intake of 100% FJ in Brazil (and therefore a greater intake of sugar from 100% FJ), as well as a lower intake of total sugar in Brazil (64 g/d) compared with the total sugar intakes in the U.S. (109 g/d) and UK (96 g/d) (data not shown). Of course, the Brazilian POF did not include young children (while young children were included in the U.S. and UK dietary intake surveys), and so the lower total sugar intake among the surveyed population in Brazil could very well be due to the absence of young children in the survey.

In the current analyses, the amount of 100% FJ consumed was associated with a significant decrease in the odds of being overweight or obese in UK adults, and statistically significant increases in the odds of being overweight or obese in UK children and Brazilian adults. In other observational studies conducted in the U.S. and UK, 100% FJ intake was not associated with a significant increase in the odds of being overweight or obese [3,4,5,6,22]. It is important to note that Brazilian dietary habits have changed significantly since the 2008 survey, with greater intake of “Western” foods and rising obesity. While the consumption of sweetened beverages is correlated with poor diets, 100% FJ consumption is correlated with healthier diets and higher socioeconomic status (SES) in the U.S., with greater intakes of fruits and vegetables and lower intakes of fatty and added sugar foods [22,23,24,25]. Consumption of 100% FJ is also correlated with higher SES in Brazil, as well as healthier dietary patterns [10]. That UK adults who consumed larger amount of 100% FJ had decreased odds of being overweight or obese is interesting. Of note, even when energy intake is removed from the multiple logistic regression model, the relationship persists. An important difference in the analyses presented herein and those conducted by others is that we did not compare BMI status among consumers and non-consumers of 100% FJ; rather, we assessed whether the amount of 100% FJ consumed was significantly associated with the odds of being overweight or obese. This analysis may have greater sensitivity, especially when energy intake is not included in the model, since large intakes of any food *should* contribute to increased odds of being overweight or obese.

Future studies should focus on how various dietary patterns associated with 100% FJ intake impact BMI, which may indicate the importance of the whole diet in weight maintenance, rather than focusing on single food groups or macronutrients. A weakness of the present study is that 100% FJ intake was not assessed in relation to lifestyle variables such as physical activity, education, or intake of other food groups. Another weakness is that the data analyzed are cross-sectional in nature, and correlations do not imply causation. However, the datasets are large, robust, and nationally representative.

In conclusion, 100% FJ contributes to micronutrient intakes in consumers. Increased 100% FJ intake was not associated with the odds of being overweight or obese in U.S. adults; however, increased 100% FJ intake was associated with a significant increase in the odds of being overweight or obese in adults in Brazil, and a significant decrease in the odds of being overweight or obese in adults in the UK. Among UK children, increased 100% FJ consumption was associated with a significant increase in the odds of being overweight or obese; no such associations were apparent among U.S. or Brazilian children.

## Figures and Tables

**Table 1:** Mean intakes of 100% FJ and contribution of 100% FJ to daily nutrient intakes in individuals reporting the consumption of 100% FJ in the U.S., UK, and Brazil.

Nutritional Variable	United States				United Kingdom				Brazil			
	Intake from 100% FJ	SEM	% of Total Intake	SEM	Intake from 100% FJ	SEM	% of Total Intake	SEM	Intake from 100% FJ	SEM	% of Total Intake	SEM
**Amount (g/d)**	183.9	4.0	N/A	N/A	130.4	5.1	N/A	N/A	248.8	3.3	N/A	N/A
**Energy (calories)**	87.9	2.0	4.7	0.1	49.7	2.0	3.0	0.1	108.7	1.5	6.2	0.1
**Total Sugars (g)**	17.0	0.5	15.0	0.3	12.5	0.5	12.1	0.5	21.1	0.3	30.7	0.3
**Vitamin C (mg)**	51.4	1.1	36.7	0.6	33.0	1.4	20.8	2.4	267.8	10.4	54.3	0.4
**Vitamin A (mcg)**	2.7	0.2	0.7	0.1	5.5	0.5	1.4	0.2	31.7	0.7	12.1	0.2
**Folate** **(mcg)**	20.0	0.8	4.3	0.2	22.3	1.0	8.4	0.5	41.1	0.6	15.2	0.2
**Potassium (mg)**	267.9	5.0	10.1	0.2	180.4	7.6	6.6	0.3	440.3	6.1	16.6	0.2
**Calcium (mg)**	77.8	3.6	7.1	0.4	12.8	0.6	1.8	0.1	20.0	0.3	5.0	0.1
**Magnesium (mg)**	16.3	0.3	6.1	0.1	10.1	0.4	4.2	0.2	29.0	0.4	12.0	0.1

**Table 2:** BMI classifications among individuals reporting 100% FJ consumption.

Survey	Number of Individuals		Body Weight Classification (number (%))					
	Reporting Intake of 100% FJ	With Complete Data (BMI and Covariates)	UW	NW	UW or NW	OW	OB	OW or OB
Children (2 to 19 years for U.S. and UK; 10 to 19 years for Brazil)								
U.S. NHANES (2013–2014)	1474	1280	47 (3.7%)	777 (60.7%)	**824** **(64.4%)**	240 (18.8%)	216 (16.9%)	**456** **(35.6%)**
UK NDNS (2012–2014)	629	579	NA	NA	**411** **(71.0%)**	85 (14.7%)	83 (14.3%)	**168** **(29.0%)**
Brazil POF (2008–2009)	3267	3267	108 (3.3%)	2455 (75.1%)	**2563** **(78.5%)**	582 (17.8%)	122 (3.7%)	**704** **(21.5%)**
Adults (20 years+)								
U.S. NHANES (2013–2014)	1404	1394	29 (2.1%)	380 (27.3%)	**409** **(29.3%)**	470 (33.7%)	515 (36.9%)	**985** **(70.7%)**
UK NDNS (2012–2014)	398	377	6 (1.6%)	155 (41.1%)	**161** **(42.7%)**	136 (36.1%)	80 (21.2%)	**216** **(57.3%)**
Brazil POF (2008–2009)	10,432	10,432	256 (2.5%)	5082 (48.7%)	**5338** **(51.2%)**	3546 (34.0%)	1548 (14.8%)	**5094** **(48.8%)**

BMI Classifications of Individuals Reporting 100% FJ Consumption, based on the 2013–2014 U.S. NHANES, 2012–2014 UK NDNS, and 2008–2009 POF. It should be noted that for the UK NDNS, children with a BMI <85th percentile are coded as “1”; therefore, it is not possible to distinguish the proportion of children who are underweight (typically defined as a BMI <5th percentile), as they are captured collectively with children who are normal weight. “NW” indicates “normal weight”; “OB” indicates “obese”; “OW” indicates “overweight”; and “UW” indicates “underweight”; bolded values represent the combinations used in the analyses (underweight or normal weight versus overweight or obese).

**Table 3:** The odds of being overweight or obese (versus underweight or normal weight) with the consumption of 100% FJ in individuals reporting the consumption of 100% FJ in the U.S. National Health and Nutrition Examination Survey (NHANES) (2013–2014): Results of multiple logistic regression models.

	Children (2 to 19 y)				Adults (20 y+)			
	With Energy Included in the Model		Without Energy Included in the model		With Energy Included in the Model		Without Energy Included in the model	
Variable	OR (95% CI)	*p*-value	OR (95% CI)	*p*-value	OR (95% CI)	*p*-value	OR (95% CI)	*p*-value
Amount of 100% FJ Consumed (per 10^2^ g/d)	1.04 (0.92, 1.17)	0.55	1.02 (0.91, 1.15)	0.68	0.95 (0.84, 1.07)	0.35	0.94 (0.84, 1.06)	0.30
Age (per 10^1^ years)	1.54 (0.91, 2.59)	0.10	1.03 (0.98, 1.09)	0.21	**1.17** **(1.01, 1.36)**	**0.04**	**1.02** **(1.00, 1.03)**	**0.03**
Gender (male versus female)	1.34 (9.85, 2.12)	0.19	1.27 (0.85, 1.89)	0.23	**1.48** **(1.02, 2.14)**	**0.04**	**1.42** **(1.00, 2.02)**	**0.051**
Race (non-Hispanic white versus others)	**0.69** **(0.50, 0.95)**	**0.02**	**0.68** **(0.50, 0.93)**	**0.02**	0.95 (0.59, 1.51)	0.80	0.94 (0.59, 1.48)	0.77
Ratio of family income to poverty	0.90 (0.78, 1.05)	0.18	0.91 (0.78, 1.06)	0.19	0.91 (0.81, 1.01)	0.08	0.90 (0.81, 1.01)	0.08
Energy (per 10^3^ kcal/d)	0.85 (0.57, 1.27)	0.40	NA	NA	0.93 (0.76, 1.12)	0.41	NA	NA

CI = confidence intervals; FJ = fruit juice; NA = not applicable; NHANES = National Health and Nutrition Examination Survey; OR = odds ratio; U.S. = United States; y = years; Bolded values were statistically significant.

**Table 4:** The odds of being overweight or obese (versus underweight or normal weight) with the consumption of 100% FJ in individuals reporting the consumption of 100% FJ in the UK NDNS (2012–2014): Results of multiple logistic regression models.

	Children (2 to 19 y)				Adults (20 y+)			
	With Energy Included in the Model		Without Energy Included in the model		With Energy Included in the Model		Without Energy Included in the model	
Variable	OR (95% CI)	*p*-value	OR (95% CI)	*p*-value	OR (95% CI)	*p*-value	OR (95% CI)	*p*-value
Amount of 100% FJ Consumed (per 10^2^ g/d)	**1.16** **(1.01, 1.33)**	**0.03**	**1.16** **(1.004, 1.33)**	**0.04**	**0.79** **(0.63, 0.99)**	**0.04**	**0.79** **(0.64, 0.98)**	**0.03**
Age (per 10^1^ years)	0.70 (0.35, 1.41)	0.32	0.66 (0.37, 1.16)	0.15	**1.34** **(1.15, 1.57)**	**0.0003**	**1.35** **(1.15, 1.58)**	**0.0003**
Gender (male versus female)	1.40 (0.85, 2.30)	0.18	1.37 (0.87, 2.14)	0.17	1.19 (0.73, 1.93)	0.48	1.11 (0.75, 1.64)	0.60
Race (white versus others)	0.997 (0.57, 1.73)	0.99	0.92 (0.58, 1.70)	0.97	**0.53** **(0.31, 0.92)**	**0.03**	**0.52** **(0.30, 0.91)**	**0.02**
Equivalised household income (per 10^4^)	**0.88** **(0.78, 0.98)**	**0.03**	**0.88** **(0.78, 0.98)**	**0.02**	1.02 (0.92, 1.12)	0.78	1.01 (0.92, 1.12)	0.79
Energy (per 10^3^ kcal/d)	0.87 (0.47, 1.62)	0.65	NA	NA	0.87 (0.51, 1.48)	0.60	NA	NA

CI = confidence intervals; FJ = fruit juice; NA = not applicable; NDNS = National Diet and Nutrition Survey; OR = odds ratio; UK = United Kingdom; y = years; Bolded values were statistically significant.

**Table 5:** The odds of being overweight or obese (versus underweight or normal weight) with the consumption of 100% FJ in Brazilians reporting the consumption of 100% FJ in the Brazil POF (2008–2009): results of multiple logistic regression models.

	Children (10 to 19 y)				Adults (20 y+)			
	With Energy Included in the Model		Without Energy Included in the model		With Energy Included in the Model		Without Energy Included in the model	
Variable	OR (95% CI)	*p*-value	OR (95% CI)	*p*-value	OR (95% CI)	*p*-value	OR (95% CI)	*p*-value
Amount of 100% FJ Consumed (per 10^2^ g/d)	0.99 (0.93, 1.06)	0.85	0.99 (0.93, 1.05)	0.74	**1.04** **(1.00, 1.09)**	**0.04**	1.04 (1.00, 1.08)	0.07
Age (per 10^1^ years)	**0.38** **(0.22, 0.68)**	**0.001**	**0.38** **(0.21, 0.68)**	**0.001**	**1.29** **(1.23, 1.36)**	**<0.0001**	**1.30** **(1.24, 1.37)**	**<0.0001**
Gender (male versus female)	**1.40** **(1.06, 1.85)**	**0.02**	**1.38** **(1.04, 1.84)**	**0.03**	**1.44** **(1.24, 1.68)**	**<0.0001**	**1.40** **(1.21, 1.62)**	**<0.0001**
Race (white versus others)	1.20 (0.90, 1.60)	0.22	1.20 (0.90, 1.60)	0.21	1.03 (0.91, 1.18)	0.61	1.04 (0.91, 1.18)	0.58
Total Family Income (per 10^4^ R$)	**2.22** **(1.57, 3.14)**	**<0.0001**	**2.19** **(1.55, 3.11)**	**<0.0001**	1.04 (0.88, 1.23)	0.63	1.04 (0.88, 1.23)	0.66
Energy (per 10^3^ kcal/d)	0.95 (0.79, 1.14)	0.57	NA	NA	0.92 (0.83, 1.03)	0.16	NA	NA

CI = confidence intervals; FJ = fruit juice; NA = not applicable; OR = odds ratio; POF = Pesquisa de Orçamentos Familiares; y = years; Bolded values were statistically significant.

## Supplementary Materials

The following are available online at https://www.mdpi.com/2072-6643/12/5/1258/s1, Table S1: 100% fruit juice food code list for the U.S., Table S2: 100% fruit juice food code list for the UK, Table S3: 100% fruit juice food code list for Brazil, Table S4: Frequency of 100% FJ Intake and Amount of 100% FJ Consumed: Results from NHANES 2013–2014.
