# Supplementary material for: Contribution of 100% Fruit Juice to Micronutrient Intakes in the United States, United Kingdom and Brazil
Source: Nutrients. 2020 Apr 28;12(5):1258. doi: 10.3390/nu12051258 (PMC7282014; doi:10.3390/nu12051258)
Supplement: Supplementary file 1 [file nutrients-12-01258-s001.pdf]

Supplementary Materials:

Supplementary Table S1: 100% fruit juice food code list for the U.S.

Food code	Description
61201010	Grapefruit juice, 100%, freshly squeezed
61201020	Grapefruit juice, 100%, NS as to form
61201220	Grapefruit juice, 100%, canned, bottled or in a carton
61201225	Grapefruit juice, 100%, with calcium added
61201620	Grapefruit juice,100%, frozen, reconstituted
61204000	Lemon juice, 100%, NS as to form
61204010	Lemon juice, 100%, freshly squeezed
61204200	Lemon juice, 100%, canned or bottled
61207000	Lime juice, 100%, NS as to form
61207010	Lime juice, 100%, freshly squeezed
61207200	Lime juice, 100%, canned or bottled
61210000	Orange juice, 100%, NFS
61210010	Orange juice, 100%, freshly squeezed
61210220	Orange juice, 100%, canned, bottled or in a carton
61210250	Orange juice, 100%, with calcium added, canned, bottled or in a carton
61210620	Orange juice, 100%, frozen, reconstituted
61210720	Orange juice, 100%, frozen, not reconstituted
61210820	Orange juice, 100%, with calcium added, frozen, reconstituted
61213220	Tangerine juice, 100%
61213800	Fruit juice blend, citrus, 100% fruit juice
61213900	Fruit juice blend, citrus, 100% fruit juice, with calcium added
64100100	Fruit juice, NFS
64100110	Fruit juice blend, 100% fruit juice
64100200	Cranberry juice blend, 100% fruit juice

64100220	Cranberry juice blend, 100% fruit juice, with calcium added
64101010	Apple cider
64104010	Apple juice, 100%
64104030	Apple juice, 100%, with calcium added
64104600	Blackberry juice, 100%
64105400	Cranberry juice, 100%, not a blend
64116020	Grape juice, 100%
64116060	Grape juice, 100%, with calcium added
64120010	Papaya juice, 100%
64121000	Passion fruit juice, 100%
64124020	Pineapple juice, 100%
64126000	Pomegranate juice, 100%
64132010	Prune juice, 100%
64132500	Strawberry juice, 100%
64133100	Watermelon juice, 100%
67202000	Apple juice, baby food
67202010	Apple juice, with added calcium, baby food
67203000	Apple-fruit juice blend, baby food
67203200	Apple-banana juice, baby food
67203400	Apple-cherry juice, baby food
67203500	Apple-grape juice, baby food
67203600	Apple-peach juice, baby food
67203700	Apple-prune juice, baby food
67203800	Grape juice, baby food
67204000	Mixed fruit juice, not citrus, baby food
67204100	Mixed fruit juice, not citrus, with added calcium, baby food
67205000	Orange juice, baby food

67211000	Orange-apple-banana juice, baby food
67212000	Pear juice, baby food

Supplementary Table S2: 100% fruit juice food code list for the UK.

Food Code	Food Description
2317	APPLE JUICE UNSWEETENED CANNED
2318	APPLE JUICE UNSWEETENED CARTONS PASTEURISED
2319	APPLE JUICE UNSWEETENED UHT
2328	GRAPEFRUIT JUICE UNSWEETENED CANNED
2329	GRAPEFRUIT JUICE UNSWEETENED PASTEURISED
2330	GRAPEFRUIT JUICE UNSWEETENED UHT
2336	ORANGE JUICE UNSWEETENED CANNED
2337	ORANGE JUICE UNSWEETENED PASTURISED
2338	ORANGE JUICE UNSWEETENED AMBIENT / UHT
2339	ORANGE JUICE FRESHLY SQUEEZED JUICE
2343	PINEAPPLE JUICE UNSWEETENED CANNED
2344	PINEAPPLE JUICE UNSWEETENED PASTEURISED
2345	PINEAPPLE JUICE UNSWEETENED UHT
8604	MANGO JUICE FRESH
8640	PRUNE JUICE BOTTLED UNSWEETENED
9350	REDCURRANT JUICE FRESH
10209	BLUEBERRY, APPLE AND GRAPE FRUIT JUICE 100% FRUIT JUICE
10285	APPLE AND BLACKCURRANT FRUIT JUICE, NOT FROM CONCENTRATE
10366	ORANGE JUICE PASTEURISED FORTIFIED WITH CALCIUM
8114	POMEGRANATE JUICE PURCHASED FORTIFIED
8450	MIXED FRUIT JUICE CANNED UNSWEETENED 100% FRUIT JUICE
9186	APPLE/PEAR JUICE CONCENTRATE UNSWEETENED
10483	VITAFIT MULTIVITAMIN 11 FRUIT JUICE

2357	MIXED FRUIT JUICE PASTEURISED
2359	FROZEN ORANGE JUICE CONCENTRATE
2360	FROZEN ORANGE JUICE CONCENTRATE MADE UP
8082	FRUIT JUICE FORTIFIED WITH MULTIVITAMINS
7892	BABY PURE FRUIT JUICE CONCENTRATE FORTIFIED WITH VITAMIN C
2096	PASSION FRUIT RAW JUICE ONLY
2132	POMEGRANATE RAW JUICE ONLY
4359	INFANT AND TODDLER PURE FRUIT JUICE WITH ADDED VITAMIN C,

Supplementary Table S3: 100% fruit juice food code list for Brazil.

Food Code	Description (Brazilian)	Description (English Translation)
8507901	SUCO ORGANICO	Orange, juice, fresh
8507907	SUCO DE LARANJA ORGANICO	Orange, juice, fresh
8500401	SUCO	Orange, juice, fresh
8500402	SUCO DE ABACAXI	Pineapple, juice, plain, purchased ready-to-drink
8500403	SUCO DE ACEROLA	Acerola juice
8500407	SUCO DE LARANJA	Orange, juice, fresh
8500408	SUCO DE LARANJA COM BANANA	Orange, juice, orange-banana
8500412	SUCO DE MAMAO	Papaya juice
8500414	SUCO DE MARACUJA	Passion fruit, juice
8500415	SUCO DE MELAO	Watermelon juice
8500416	SUCO DE MORANGO	Strawberry juice
8507902	SUCO DE ABACAXI ORGANICO	Pineapple, juice, plain, purchased ready-to-drink
8507903	SUCO DE ACEROLA ORGANICO	Acerola juice
8507914	SUCO DE MARACUJA ORGANICO	Passion fruit, juice
8507916	SUCO DE MORANGO ORGANICO	Strawberry juice

Supplementary Table S4: Frequency of 100% FJ Intake and Amount of 100% FJ Consumed: Results from NHANES 2013-2014.

Population Group (age, years)	Consumption of 100% juice on EITHER day of the 2-day survey				Consumption of 100% juice on ONLY Day 1 of the 2-day survey				Consumption of 100% juice on BOTH days of the 2-day survey (BOTH Days 1 and 2)			
	Number	% users (of total surveyed)	Mean Intake (g/d)	SEM (g/day)	Number	% of total users	Mean Intake (g/d)	SEM (g/day)	Number	% of total users	Mean Intake (g/d)	SEM (g/day)
Children (<1.5)	123	51.85	139.14	15.80	29	32.72	136.17	20.58	73	47.63	205.99	20.10
Children (1.5 to 2)	159	66.76	177.73	12.20	40	22.62	181.65	23.50	84	50.59	243.98	17.82
Children (3 to 9)	599	59.42	193.08	8.51	148	27.34	216.99	10.92	279	44.15	267.63	18.94
Children (10 to 12)	227	47.84	181.85	13.27	59	28.17	221.86	13.57	76	33.17	273.93	21.36
Male Teens (13 to 19)	189	41.42	222.60	28.82	71	35.65	375.62	32.60	46	21.95	428.83	58.27
Female Teens (13 to 19)	177	26.44	165.44	15.37	64	33.72	285.85	42.80	38	21.62	287.91	50.84
Male Young Adults	416	26.08	217.16	12.82	155	34.58	323.40	27.00	103	29.32	332.24	33.69

(20 to 59)												
Female Young Adults (20 to 59)	476	28.96	161.67	10.53	182	37.18	204.08	11.47	111	25.17	307.32	29.17
Male Older Adults (60+)	230	31.72	182.39	11.82	66	29.81	226.20	18.96	93	45.75	272.11	13.33
Female Older Adults (60+)	282	34.92	158.45	14.02	71	25.83	177.18	20.20	113	45.54	207.08	14.50
Total Population (0+)	2878	34.16	183.87	3.97	885	31.74	243.71	7.93	1016	34.61	280.69	8.57
